# Long-term neurodevelopmental outcomes of significant neonatal jaundice in Taiwan from 2000–2003: a nationwide, population-based cohort study

**DOI:** 10.1038/s41598-020-68186-w

**Published:** 2020-07-09

**Authors:** Pei-Chen Tsao, Hsin-Ling Yeh, Yu-Shih Shiau, Yen-Chen Chang, Szu-Hui Chiang, Wen-Jue Soong, Mei-Jy Jeng, Kwang-Jen Hsiao, Po-Huang Chiang

**Affiliations:** 1 Division of Neonatology and Critical Care, Pediatrics Department, Taipei Veterans General Hospital, No. 201, Sec. 2, Shipai Rd., Beitou District, Taipei City, 1217 Taiwan, ROC; 2 Department of Pediatrics, School of Medicine, National Yang-Ming University, Taipei City, Taiwan, ROC; 3 Institute of Physiology, School of Medicine, National Yang-Ming University, Taipei City, Taiwan, ROC; 4 Institute of Emergency and Critical Care Medicine, School of Medicine, National Yang-Ming University, Taipei City, Taiwan, ROC; 5 The Institute of Population Health Sciences, National Health Research Institutes, 35 Keyan Road, Zhunan, Miaoli County, 35053 Taiwan, ROC; 6 Preventive Medicine Foundation, Taipei Xinwei P.O. Box 26-624, Taipei City, 10699 Taiwan, ROC

**Keywords:** Diseases, Health care, Medical research

## Abstract

Newborns with significant neonatal jaundice (SNJ) would admit for evaluation and/or intervention due to an earlier or more rapid increase in bilirubin level. Bilirubin-induced neurological dysfunction in this population might be underestimated. We aimed to investigate the risk of long-term neurodevelopmental sequelae of SNJ in Taiwan. An SNJ 2000–2003 follow-up cohort consisting of 66,983 neonates was extracted from the nationwide, population-based health insurance database in Taiwan to survey the accumulative incidence of long-term (7-year) neurodevelopmental sequelae in comparison to a reference general-population neonate cohort of 12,579 individuals born in 2000. The SNJ follow-up cohort was furtherly categorized into subgroups according to interventions (phototherapy, intensive phototherapy, and exchange transfusion). The SNJ follow-up cohort exhibited significantly higher cumulative rates of long-term neurodevelopmental sequelae than did the reference cohort (*P* < 0.05). The risks of infantile cerebral palsy, hearing loss, and developmental delay in the SNJ follow-up cohort were between twice and three times of those in the reference cohort after adjusting for gender, comorbid perinatal disorders and urbanization levels. All intervention subgroups demonstrated higher risks for long-term neurodevelopmental sequelae than the reference cohort (*P* < 0.05) after adjustment. Patients with SNJ are at risk of developing neurodevelopmental disorders during their growth period. A scheduled follow-up protocol of physical and neurodevelopmental assessment during early childhood for these SNJ patients would potentially be helpful for the early detection of and intervention for neurodevelopmental disorders.

## Introduction

Unlike physiological jaundice, newborns with significant neonatal jaundice (NJ) have an earlier and/or more rapid increase in bilirubin level. These cases would need admission because their bilirubin level met with the criteria for intervention and further evaluation^1^. The greatest concern regarding significant neonatal jaundice (SNJ) is bilirubin-associated neurotoxicity caused by the spreading of unconjugated bilirubin across the immature blood–brain barrier. Growing evidence suggests that in addition to kernicterus and death, neurological dysfunctions (a spectrum of neurological manifestations) can result from taking bilirubin and are underrepresented in medical research, which indicates that more attention must be paid to treating infants exposed to mild degrees of neonatal hyperbilirubinemia^2–4^. Thus far, several studies investigating potential associations between neonatal hyperbilirubinemia and neurodevelopmental disorders have been either cross-sectional studies or cohort studies with relatively small numbers of patients^5–7^. To overcome these limitations, longitudinal studies using relatively large populations with precise diagnoses by board-certificated psychiatrists are needed to validate the negative impact of SNJ on the development of neurodevelopmental disorders.

The incidence of referral for NJ was reported around 29.3–39.7 per 1,000 live births^8,9^. The respective average incidence and mortality rates of SNJ in Taiwan were 7.1% (5.86–8.46%) and 0.39% (0.29–0.51%) between 2000 and 2003^10^. When compared with previous data collected in Taiwan^11^, a dramatic reduction in the number of incidences of acute severe sequelae caused by SNJ can be observed^10^. However, data regarding the long-term neurodevelopmental outcomes associated with SNJ in Taiwan is lacking. The health burden of Bilirubin-induced neurological dysfunction might be underestimated or ignored. In this study, we used a nationwide population-based dataset to conduct a longitudinal cohort study. The aim was to determine whether SNJ increased the risk for neurodevelopmental disorders in children.

## Results

**Figure 1:**
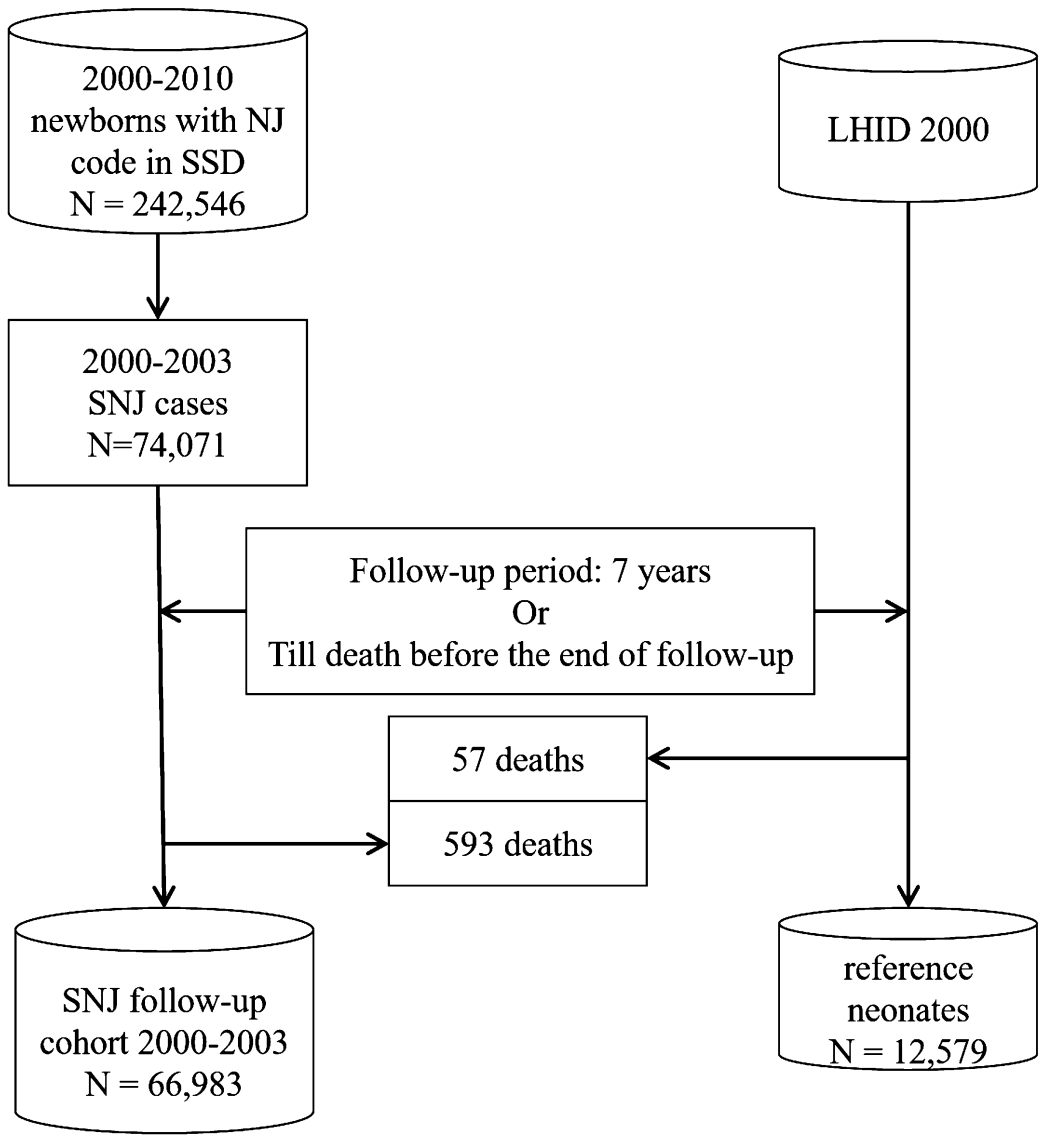
Flowchart of significant neonatal jaundice (SNJ) occurrences in the 2000–2003 follow-up cohort and 2000 reference cohort. Abbreviations: LHID 2000, Longitudinal Health Insurance Database 2000; SSD, Specific Subject Dataset; NJ, neonatal jaundice.

**Figure 2:**
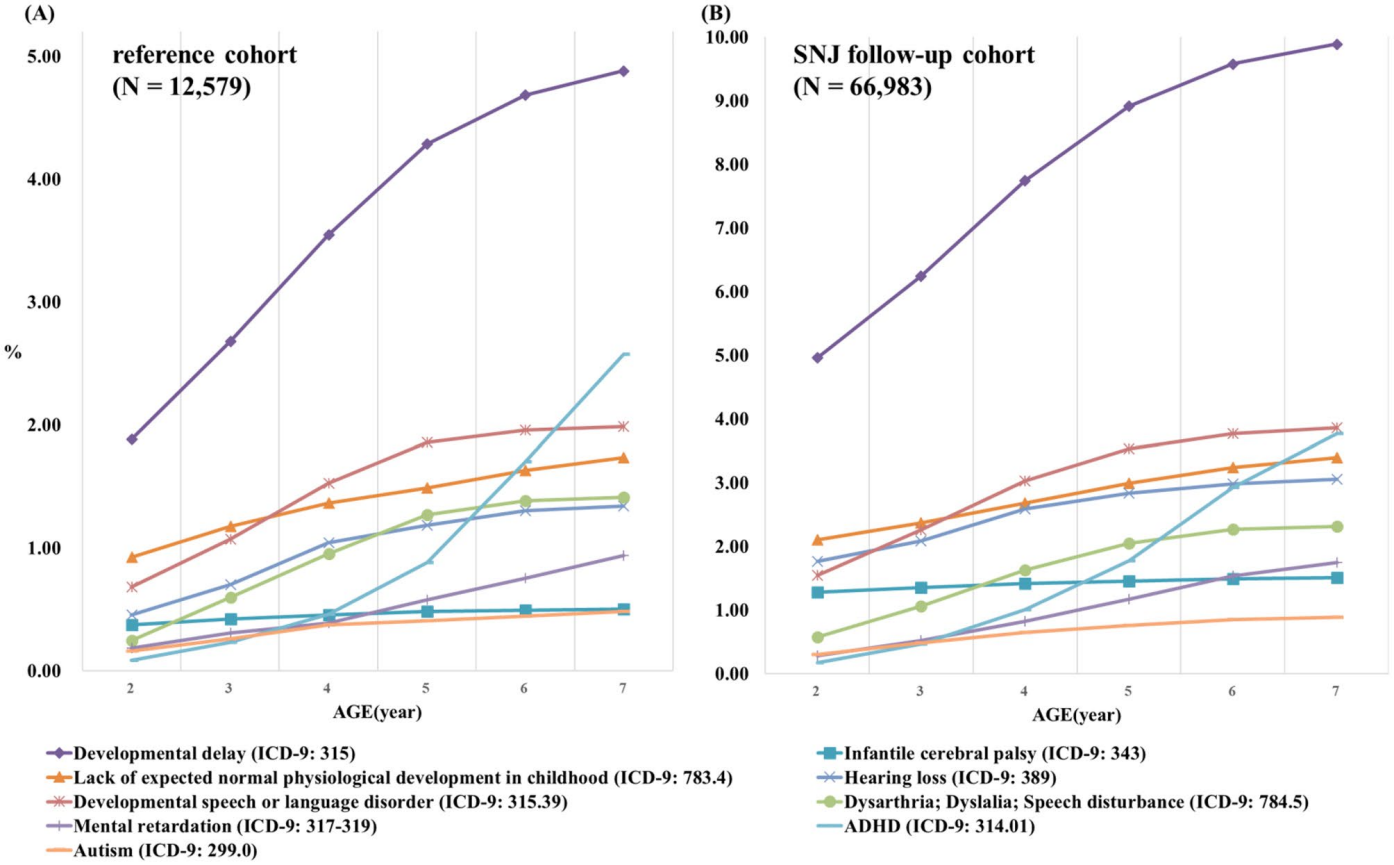
The cumulative incidence of long-term sequelae (ICD-9-CM) in (**A**) the reference cohort and (**B**) the SNJ follow-up cohort.

**Figure 3:**
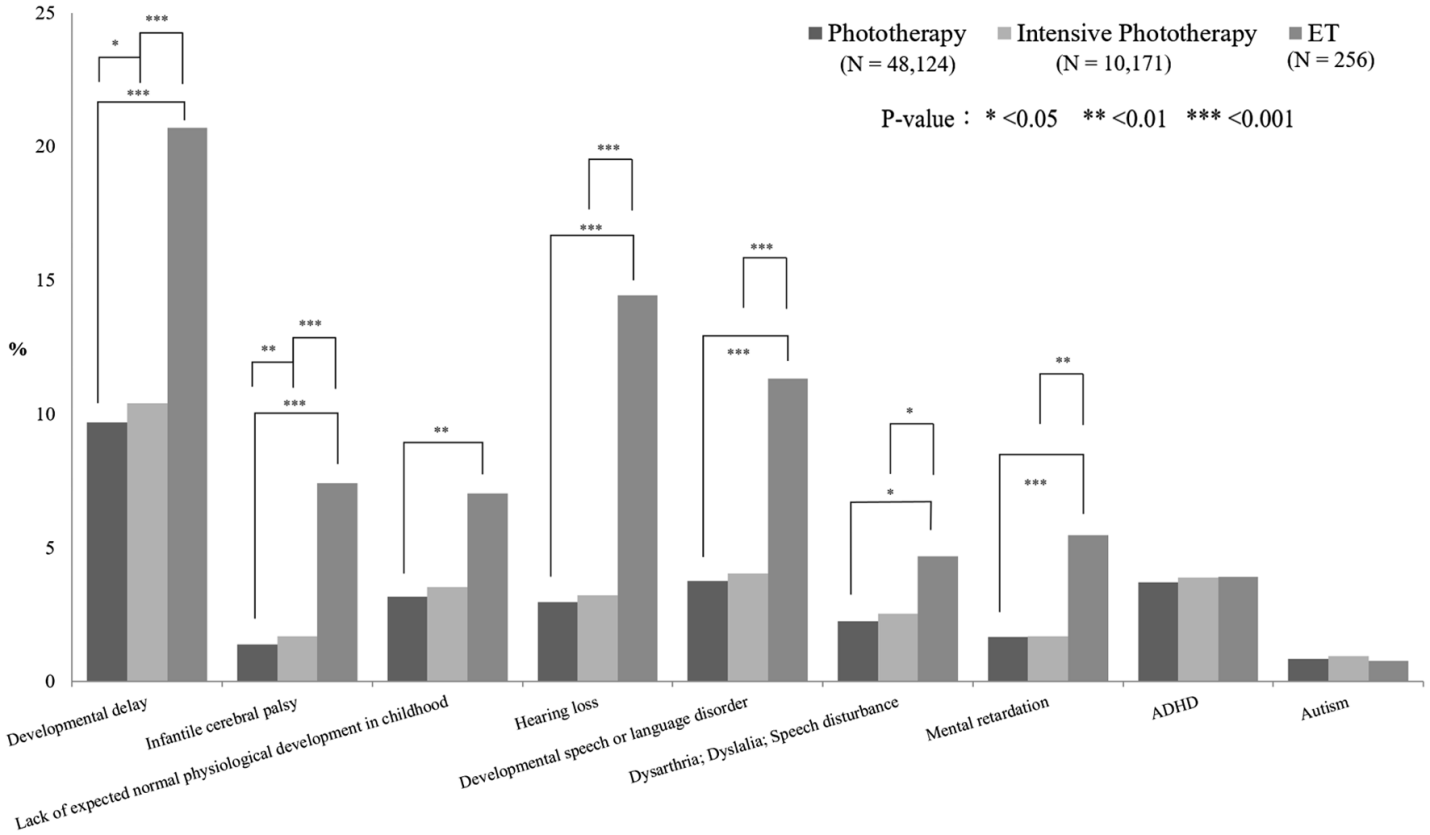
The cumulative incidence of long-term neurodevelopmental sequelae for various intervention subgroups. ^#^P was calculated using multivariate logistic regression.

An SNJ 2000–2003 follow-up cohort consisting of 66,983 neonates who hospitalized with NJ code were selected from the specific subject dataset (SSD) of the National Health Insurance Research Database (NHIRD) from 2000 to 2003 (Fig. 1)^10^. Most of the enrolled newborns (98.4%) admitted with an NJ diagnosis within their first 14 days of life (data not shown). There were 57 and 593 deaths in the reference cohort (12,579 neonates in Longitudinal Health Insurance Database 2000 (LHID 2000), Fig. 1) and SNJ cohort, respectively. According to the most advance treatment option in the individual records, there were 48,124 (71.8%), 10,171 (15.2%), and 256 (0.38%) cases re-grouping into subgroups of simple phototherapy, intensive phototherapy, and exchange transfusion (ET), respectively. Of SNJ 2000–2003 follow-up cohort, 8,432 cases received no treatment. Both the 2000–2003 SNJ follow-up cohort and the reference cohort exhibited a similar trend in cumulative incidence rates of long-term neurodevelopmental sequelae, most of which plateaued when patients reached the age of 7 years (Fig. 2). Therefore, this study conducted follow-ups for all subjects in these two cohorts until patients reached the age of 7 years or until death happening before the end of follow-up (Fig. 1). Table 1 presents the clinical characteristics of the two study cohorts. The number of males in the 2000–2003 SNJ follow-up cohort (58.0%) was significantly higher than that in the reference cohort (51.6%) (*P* < 0.001). The characteristics and demographic data are presented in Table 1. SNJ 2000–2003 follow-up cohort exhibited higher incidence rates of perinatal comorbidities at birth, including preterm births, hemolytic disease of the fetus or newborn, and congenital chromosome anomalies, than did the reference cohort (*P* < 0.001, Table 1). There was also significant difference of urbanization levels between two cohorts (*P* < 0.001). The age of patients at first diagnosis of sequelae, including developmental delay, hearing loss, developmental speech disorder, and ADHD, was significantly younger in the 2000–2003 SNJ follow-up cohort than in the reference cohort (*P* < 0.05, data not shown). The 2000–2003 SNJ follow-up cohort exhibited significantly higher cumulative rates of these sequelae than did the reference cohort (*P* < 0.05, Table 2). Subjects in the 2000–2003 SNJ follow-up cohort were at 1.5–3 times the risk of developing these long-term neurodevelopmental sequelae compared with those in the reference cohort (Table 2), which was similar to findings for the subgroups of the 2000–2003 SNJ follow-up cohort regarding phototherapy and intensive phototherapy (Table 3). Our results indicated double to triple the risk of infantile cerebral palsy, hearing loss, and developmental delay in the 2000–2003 SNJ follow-up cohort and its subgroups compared with the reference cohort. In the 2000–2003 SNJ follow-up cohort, patients treated with exchange transfusion also exhibited significantly higher risks for these long-term sequelae, except for attention-deficit-hyperactivity disorder (ADHD) and autism spectrum disorder (ASD), than did those in the reference cohort (Table 3). After adjusting for gender, urbanization levels, and comorbid perinatal disorders, including preterm births, hemolytic disease of the fetus or newborn, and congenital or chromosome anomalies, the risk for these neurodevelopmental sequelae remained higher in the 2000–2003 SNJ follow-up cohort than in the reference cohort (Table 2). Compared with the reference group, the simple phototherapy subgroup demonstrated higher risks for long-term sequelae development, except for infantile cerebral palsy (CP) and mental retardation, after making adjustments for sex, urbanization levels, and comorbid perinatal disorders (Table 3). Similarly, the intensive phototherapy subgroup exhibited higher risks for long-term sequelae, except mental retardation, than did the reference cohort (Table 3). Figure 3 presents the results of between-group analyses for various subgroups. Except for ADHD and ASD, ET subgroup had higher cumulative incidence rates of long-term sequela development than did either of the phototherapy subgroups (*P* < 0.05; Fig. 3), even after adjusting for gender, urbanization levels, and comorbid perinatal disorders. Moreover, higher cumulative incidence rates for developmental delay, infantile CP, hearing loss, development speech disorders, and mental retardation were found for the ET subgroup than for the intensive phototherapy subgroup (*P* < 0.01; Fig. 3). Patients in this cohort who underwent intensive phototherapy had significantly higher cumulative incidence rates of developmental delay and infantile CP than those who only underwent phototherapy (*P* < 0.05; Fig. 3).

**Table 1:** Demographic data of the two study cohorts.

	Reference cohort 2000 (n = 12,579)	SNJ follow-up cohort 2000–2003 (n = 66,983)	*P*
**Gender (n, %)**			
Male	6,496 (51.6)	38,861 (58.0)	< 0.001
**Comorbid perinatal conditions (n, %)**			
Preterm	414 (3.3)	15,636 (23.3)	< 0.001
Hemolytic disease of fetus and newborn	38 (0.3)	1,730 (2.6)	< 0.001
Congenital or chromosome anomalies	2,039 (16.2)	18,543 (27.7)	**< **0.001
**Urbanization (n, %)**			
Level 1-Highly urbanized cities	8,152 (64.81)	50,274 (75.05)	**< **0.001
Level 2-Moderate urbanized cities	3,799 (30.2)	15,570 (23.24)	**< **0.001
Level 3-Emerging cities	452 (3.59)	660 (0.99)	**< **0.001
Level 4-Other	120 (0.95)	425 (0.63)	**< **0.001

**Table 2:** Comparison of risks of long-term sequelae between the SNJ cases and reference cohort.

Different categories	Reference cohort (n = 12,579)	SNJ 2000–2003 follow-up cohort (n = 66,983)		
	n (%)	n (%)	OR	aOR^a^
Developmental delay	614 (4.9)	6,630 (9.9)	2.14*	1.63***
Infantile cerebral palsy	63 (0.5)	1,009 (1.5)	3.03*	1.35*
Lack of expected normal physiological development in childhood	218 (1.73)	2,277 (3.4)	1.99*	1.5***
Hearing loss	169 (1.3)	2,047 (3.1)	2.31*	1.74***
Developmental speech disorders	250 (2.0)	2,587 (3.9)	1.98*	1.51***
Dysarthria	178 (1.4)	1,553 (2.3)	1.65*	1.46***
Mental retardation	118 (0.9)	1,169 (1.8)	1.87*	1.27*
ADHD	324 (2.58)	2,523 (3.8)	1.48*	1.3***
Autism	61 (0.5)	593 (0.9)	1.83*	1.69***

*SNJ* significant neonatal jaundice, *ADHD* attention deficit hyperactivity disorder.

*P 0.05; **P 0.01; ***P 0.001.

^a^Adjusted for gender, urbanization levels and comorbid perinatal conditions (prematurity, hemolytic disease of newborn, and congenital or chromosome anomalies).

**Table 3:** Comparison of cumulative incidences and risks of long-term sequelae between SNJ cases of varying severity and the reference cohort.

Different categories	Reference neonates (n = 12,579)		SNJ (level of severity)											
			Phototherapy (n = 48,124)				Intensive phototherapy (n = 10,171)				ET (n = 256)			
	n	%	n	%	OR	aOR^a^	n	%	OR	aOR^a^	n	%	OR	aOR^a^
Developmental delay	614	4.88	4,669	9.70	1.78***	1.59***	1,058	10.40	1.94***	1.72***	53	20.70	3.37***	3.17***
Infantile cerebral palsy	63	0.50	664	1.38	1.68**	1.25	173	1.70	1.67*	1.4*	19	7.42	7.99**	5.08***
Lack of expected normal physiological development in childhood	218	1.73	1,524	3.17	1.55***	1.42***	359	3.53	1.55***	1.55***	18	7.03	3.03*	2.94***
Hearing loss	169	1.34	1,427	2.97	1.75***	1.62***	327	3.22	2.19***	1.73***	37	14.45	10.01***	5.71***
Developmental speech disorder	250	1.99	1,806	3.75	1.69***	1.51***	411	4.04	1.78***	1.63***	29	11.33	4.50***	4.5***
Dysarthria	178	1.42	1,084	2.25	1.58***	1.39***	258	2.54	1.77***	1.45***	12	4.69	3.60**	2.39*
Mental retardation	118	0.94	798	1.66	1.35**	1.2	172	1.69	1.39*	1.23	14	5.47	3.46*	2.16*
ADHD	324	2.58	1,792	3.72	1.46***	1.24***	396	3.89	1.49***	1.26**	10	3.91	2.26	1.04
Autism	61	0.48	416	0.86	1.82***	1.5**	98	0.96	2.14***	1.59**	2	0.78	1.75	0.83

*SNJ* significant neonatal jaundice, *ET* exchange transfusion, *ADHD* attention deficit hyperactivity disorder.

*P 0.05; **P 0.01; ***P 0.001.

^a^Adjusted for gender, urbanization levels, and comorbid perinatal conditions (prematurity, hemolytic disease of newborn, and congenital or chromosome anomalies).

## Discussion

Evidence from this population-based study indicated that patients in the 2000–2003 SNJ follow-up cohort had a 1.5- to 3-times greater risk for nine long-term neurodevelopmental sequelae, namely developmental delay, mental retardation, infantile CP, abnormal physiological development in childhood, developmental speech or language disorders, dysarthria, hearing loss, ADHD, and ASD, than did the reference cohort. Even after adjusting for gender, urbanization levels, and other perinatal factors which might have affected the development of SNJ or neurodevelopmental disorders, the 2000–2003 SNJ follow-up cohort and subgroups still exhibited higher risks for these long-term sequelae than did the reference cohort. Patients diagnosed with developmental delay, hearing loss, development speech disorders, or ADHD were younger in the 2000–2003 SNJ follow-up cohort than in the reference cohort.

Selective damage to gray matter in central nervous system, such as basal ganglia or thalamus, caused by bilirubin toxicity leads to neurological sequelae, including acute, chronic bilirubin encephalopathy, and bilirubin-induced neurological dysfunction^12^. However, whether moderate hyperbilirubinemia led to long-term neurological abnormalities remained inconclusive. Some studies failed to discern an association between moderate hyperbilirubinemia and impairments in general neurodevelopment^13,14^. Other studies reached a somewhat different conclusion. Full-term newborns with moderate hyperbilirubinemia, including those with no clinical sequelae at discharge, have a reportedly increased risk of minor neurological dysfunction and motor and developmental disturbances at the ages of 18 and 32 months^15^. Amin et al.^16^ and Mamidala et al.^5^ reported NJ as a prevalent neonatal risk factor for ASD. A population-based cohort study found that neonates with NJ were at high risk for physician-diagnosed ADHD during their period of growth^17^. Our results suggested that neonates with SNJ had higher risks of hearing loss, speech or language disorders, developmental disturbances, and behavior disorders than did the reference cohort. In our study, patients receiving simple phototherapy for SNJ still exhibited significant risks for these long-term neurological sequelae (*P* < 0.05), except infantile CP and mental retardation. This result might suggest that for these neonates, clinicians and parents should remain aware of the probability of neurodevelopmental disorders and focus on monitoring them for signs of such disorders. As expected, significantly higher cumulative incidences of developmental delay, infantile CP, abnormal physiological development, hearing and speech disorders, and mental retardation presented in SNJ patients treated with ET, regardless of whether they were compared with the reference cohort or one of the intervention subgroups. However, the increased risk of ADHD and ASD was found not significant in SNJ patients treated with ET, possibly because of the small sample size leading to a lack of statistical power. Cordero et al. found that neonatal jaundice was a risk factor for ASD and non-ASD developmental disorders in late-preterm subjects^18^. In this study, preterm subjects with low body weight (1500 g–2499 g) in SNJ 2000–2003 follow-up cohort had a significantly higher rate of developmental delay than those in reference cohort had (data not shown).

In the SNJ 2000–2003 follow-up cohort, most of the patients diagnosed with these neurodevelopmental sequelae were younger than those in the reference cohort (Table 1). The Health Promotion Administration of the Ministry of Health and Welfare provided seven health checks for children aged younger than 7 years in Taiwan. In this study, we discovered a cluster phenomenon of these abnormal cases detected during these routinely scheduled physical checks (data not shown). A scheduled follow-up physical examination, including neurodevelopmental assessment, during early childhood could be helpful for the early detection and intervention for these neurodevelopmental sequelae in SNJ patients.

Using longitudinal design with a large sample size, this study provided the evidence of risk for long-term neurodevelopmental disorders among children with SNJ. However, our study had several limitations. First, due to limitations inherent in the specific NHIRD data set, the peak bilirubin level that was required to clarify the severity of SNJ was lacked in this study. Pediatricians and neonatologists in Taiwan usually treat SNJ according to the clinical practice guidelines published by the American Academy of Pediatrics^19^. Therefore, we categorized the severity of SNJ according to the different interventions according to the most advanced treatment option (ET > intensive phototherapy > phototherapy) in our study. Although Taiwan National Health Insurance Administration had set up the criteria for health insurance payment according to the guideline of disease management and audited the clinicians’ judgment and management routinely, inconsistencies between physician practices is inevitable. Second, our study used the ICD-9 diagnosis code set to detect target subjects. One concern regarding ICD-9 was the lack of specificity of the information conveyed through the codes. For example, infantile CP is categorized into different detailed types, including athetoid CP and dystonia/hyperkinetic CP, and the ICD-9 code is unable to distinguish these types. The ICD-10 diagnosis code set was not implemented until January 2015 in Taiwan, and its use in future studies on the adverse long-term neurodevelopmental disorders in SNJ cases would provide more detailed and valuable data. Third, other variables, such as maternal health information, economic condition, and some comorbid diseases of preterm birth, would have impact on the neurodevelopment. Unfortunately, NHIRD lack these detailed data to eliminate these confounders. Fourth, due to the limitation and characteristics of NHIRD, the SNJ 2000–2003 follow-up cohort and reference cohort were extracted from two different databases. The sample size of reference cohort was smaller. Although the reference cohort consists of 12,579 subjects, which is only about one fifth of the subjects in SNJ 2000–2003 follow-up cohort. Further study is needed to eliminate these effects and to validate the impact of SNJ on neurodevelopmental disorders.

In conclusion, our results indicated SNJ was a potential risk factor for long-term neurodevelopmental sequelae, particularly development delay, infantile CP, and hearing deficits. However, the adverse effects of SNJ on long-term neurodevelopment have possibly been underestimated. Although continuing to reduce the occurrence of acute severe sequelae in patients is a worthwhile goal, increased research efforts should focus on both elucidating the effects of SNJ on neurodevelopment and improving its long-term outcomes. A protocol with more frequent and comprehensive evaluations and longer follow-up durations to monitor the potential development of neurodevelopment disorders in this population should be took in to consideration.

## Methods

### Databases

This retrospective, population-based study was conducted using data from the Taiwan NHIRD released by the Taiwan National Health Research Institute (NHRI)^20^. The National Health Insurance program in Taiwan was established in 1995 to provide comprehensive public healthcare; as of 2014, 99.9% of Taiwan’s population (23.74 million) was enrolled (https://www.nhi.gov.tw/). The National Health Insurance program in Taiwan offers integrated medical care, including inpatient, outpatient, emergency, dental, and traditional Chinese medical services, as well as medical prescriptions^21^. The NHRI audits and releases the NHIRD for research purposes. Personal identities are encrypted prior to the public release of the data set. The database includes comprehensive information regarding insured subjects, such as demographic data, dates of clinic visits, disease diagnoses, and interventions. The diagnostic codes of patients in the NHIRD are established by board-certified physicians in their corresponding specialties with the format of the International Classification of Diseases, Ninth Revision, Clinical Modification (ICD-9-CM). The NHRI and the institutional review board of the NHRI approved this study (IRB approval number: EC1041107-E).

### Selection of the SNJ 2000–2003 follow-up cohort

This study used a Specific Subject Dataset (SSD) comprised of medical records of neonates who hospitalized with NJ code (ICD-9-CM, 774) (n = 242,546) from all live births (n = 2,428,341) from Jan. 1, 2000 to Dec. 31, 2010^10^. From this SSD, subjects born within 31 days between Jan. 1, 2000 to Dec. 31, 2003 were extracted as SNJ 2000–2003 follow-up cohort. According to the recommendations^19^ and physicians’ judgment, these cases needed admission for intervention and further evaluation due to an abnormal process of jaundice development other than physiological jaundice. These cases were followed up until either the age of 7 years or death, whichever came first. The incidence rates of nine possible long-term neurodevelopmental sequelae, namely developmental delay (ICD-9-CM, 315), mental retardation (ICD-9-CM, 317–319), infantile cerebral palsy (ICD-9-CM, 343), lack of expected normal physiological development in childhood (ICD-9-CM, 783.4), developmental speech or language disorder (ICD-9-CM, 315.39), dysarthria (ICD-9-CM, 784.5), hearing loss (ICD-9-CM, 389), and attention deficit hyperactivity disorder (ICD-9-CM, 314.01), and autism spectrum disorders (ICD-9-CM, 299.0), were monitored in the 2000–2003 SNJ follow-up cohort. Each of these conditions was confirmed if the same diagnosis code appeared twice or more on one record. Using the procedure codes for phototherapy (ICD-9-CM, 99.83) and exchange transfusion (ICD-9-CM, 99.01), the data on SNJ interventions were also collected. The treatment codes in the NHIRD classified the phototherapy intervention as simple and intensive phototherapy. The Taiwan National Health Insurance program set up the criteria for medical interventions and audited the clinical practice routinely. Due to lack of laboratory data on medical records of every participants in NHIRD, we used the most advanced treatment option (ET > intensive phototherapy > phototherapy) to represent the bilirubin level and severity of NJ in this study. We also analyzed gender, comorbid perinatal conditions (preterm or low birth weight, ICD-9-CM code: 765; hemolytic disease of the fetus or newborn, ICD-9-CM code: 773; congenital or chromosomal anomalies, ICD-9-CM codes: 740–759), and urbanization levels^22^ for their effect on these long-term neurodevelopmental sequelae.

### Selection of reference neonates

The reference neonate cohort used in this study was selected from the LHID 2000^23^, which contains all of the original claim data of 1 million insured subjects who were selected randomly from the year 2000 in the Registry for Beneficiaries of the NHIRD (https://nhird.nhri.org.tw/en/Data_Subsets.html). The occurrence of comorbid perinatal conditions and urbanization levels in reference cohort were investigated. These reference neonates were also monitored for these nine possible long-term neurodevelopmental sequelae of SNJ.

### Statistical analysis

Continual variables were expressed as a number (*n*), proportion (%), and mean ± standard deviation. Categorical data were analyzed using Pearson’s χ^2^ when appropriate. Odds ratios (ORs) was calculated with a univariate logistic regression to analyze the risks for long-term neurodevelopmental sequelae. Multivariate logistic regression was used to calculate adjusted ORs (aORs) for comparing the 2000–2003 SNJ follow-up cohort with the reference cohort, as well as making comparisons between the subgroups within the SNJ follow-up cohort after making adjustments for gender, urbanization levels, and perinatal variables (prematurity, hemolytic disease of newborn, and congenital or chromosome anomalies). Two-tailed P-values less than 0.05 were considered statistically significant for all analyses. All data management and analyses were performed using the R programming language (Windows version 3.2; https://cran.r-project.org/).

### Ethics approval and consent to participate

IRB Approval No. EC1041107-E.

## Data Availability

The data that support the findings of this study are available from NHIRD but restrictions apply to the availability of these data, which were used under license for the current study, and so are not publicly available. Data are however available from the authors upon reasonable request and with permission of NHRI.

## Abbreviations

NJ: Neonatal jaundice
SNJ: Significant neonatal jaundice
NHIRD: National Health Insurance Research Database
NHRI: National Health Research Institute
ICD-9-CM: International Classification of Disease, Ninth Revison, Clinical Modification
SSD: Specific subject dataset
CP: Cerebral palsy
ADHD: Attention deficit hyperactivity disorder
ASD: Autism spectrum disorders
ET: Exchange transfusion
LHID: Longitudinal Health Insurance Database
aORs: Adjusted odds ratios
